# Plant Functional Groups Dominate Responses of Plant Adaptive Strategies to Urbanization

**DOI:** 10.3389/fpls.2021.773676

**Published:** 2021-11-30

**Authors:** Yihua Xiao, Shirong Liu, Manyun Zhang, Fuchun Tong, Zhihong Xu, Rebecca Ford, Tianlin Zhang, Xin Shi, Zhongmin Wu, Tushou Luo

**Affiliations:** ^1^Research Institute of Tropical Forestry, Chinese Academy of Forestry, Guangzhou, China; ^2^Research Institute of Forest Ecology, Environment and Protection, Chinese Academy of Forestry, Beijing, China; ^3^College of Resources and Environment, Hunan Agricultural University, Changsha, China; ^4^Environmental Futures Research Institute, School of Environment and Science, Griffith University, Brisbane, QLD, Australia; ^5^College of Forestry and Landscape Architecture, South China Agricultural University, Guangzhou, China

**Keywords:** urbanization, plant functional groups, maximum photosynthetic rate, heavy metal, leaf N allocation

## Abstract

Urbanization causes alteration in atmospheric, soil, and hydrological factors and substantially affects a range of morphological and physiological plant traits. Correspondingly, plants might adopt different strategies to adapt to urbanization promotion or pressure. Understanding of plant traits responding to urbanization will reveal the capacity of plant adaptation and optimize the choice of plant species in urbanization green. In this study, four different functional groups (herbs, shrubs, subcanopies, and canopies, eight plant species totally) located in urban, suburban, and rural areas were selected and eight replicated plants were selected for each species at each site. Their physiological and photosynthetic properties and heavy metal concentrations were quantified to reveal plant adaptive strategies to urbanization. The herb and shrub species had significantly higher starch and soluble sugar contents in urban than in suburban areas. Urbanization decreased the maximum photosynthetic rates and total chlorophyll contents of the canopies (*Engelhardtia roxburghiana* and *Schima superba*). The herbs (*Lophatherum gracile* and *Alpinia chinensis*) and shrubs (*Ardisia quinquegona* and *Psychotria rubra*) species in urban areas had significantly lower nitrogen (N) allocated in the cell wall and leaf δ^15^N values but higher heavy metal concentrations than those in suburban areas. The canopy and subcanopy (*Diospyros morrisiana* and *Cratoxylum cochinchinense*) species adapt to the urbanization via reducing resource acquisition but improving defense capacity, while the herb and shrub species improve resource acquisition to adapt to the urbanization. Our current studies indicated that functional groups affected the responses of plant adaptive strategies to the urbanization.

## Introduction

Urbanization, through conversions of natural or agricultural environments to urban environments and population mobility from rural to urban areas, has generated overwhelming impacts on the territorial ecological system (Sala et al., 2000; Zhao et al., 2016). Globally, the geographical areas that are influenced by urbanization are estimated to triple in size from 2000 to 2030 (Seto et al., 2012). Relative to suburban areas, high population densities and intensive anthropogenic activities in urban areas have led to marked alterations in atmospheric, soil, and hydrological environmental factors (Sala et al., 2000; Beaumont et al., 2008; Zhao et al., 2016). Urbanization also resulted in an increase in environmental pollution originating from fossil fuel burning, gas emissions of vehicles, and industrial production. At the same time, urban heat islands have caused large effects from elevated temperatures on surrounding suburbia and hinterlands (Ferguson and Woodbury, 2007; Beaumont et al., 2008). These negative effects may be decreased partially through enhancing urban vegetation surface and density. For instance, urban vegetation tremendously reduced nitrogen dioxide, particulate matter, heavy metals, and heat island effects (Qiu et al., 2009; Pugh et al., 2012; Janhäll, 2015; Livesley et al., 2016). Understanding how urban vegetation responses and adapts to the urban environment is crucial for plant species choice or selection in urban green practice.

Within the urban vegetation communities, influential ecological factors to which an individual plant must adapt include water availability, atmospheric nitrogen (N) and phosphorus (P) dispositions, and pollutant exposure. Without these adaptive abilities, an individual plant would not leave descendants or clades to flourish (Wiens et al., 2010). Plant functional types can significantly influence the leaf traits (Shi et al., 2020) and the light, heat, and N dispositions and pollutant exposure could directly affect the photosynthesis, leaf properties, plant physiology, and, thereby, the vegetation community (Quigley, 2002; Zhao et al., 2016; Liu et al., 2020). For instance, Quigley (2002, 2004) suggested that trees in urban areas grew slower than those in rural areas due to environmental stresses such as higher temperature and water limitation. Conversely, tree seedlings in New York City grew twice as fast compared to those in the rural areas (Gregg et al., 2003), and Zhao et al. (2016) also demonstrated that an urban environment accelerates vegetation growth. In addition, urbanization might affect a range of morphological and physiological plant traits, and correspondingly, the plants might adopt different strategies to adapt to the urbanization promotion or pressure (Gregg et al., 2003; Hahs and Mcdonnell, 2007; Zhao et al., 2016). However, to the best of our knowledge, few studies have been conducted to reveal the physiological responses of different plants to the urbanization.

The plants could be divided into different functional groups: herbs, shrubs, subcanopies, and canopies according to their heights and external sizes. We hypothesized that the urbanization might discrepantly affect the different fictional plant groups. Moreover, the plants could remove urban environmental pollution, and therefore, it was also hypothesized that the urbanization might exert negative impacts on the subcanopies and canopies with large external sizes. Functional trait provides an opportunity to optimize urban vegetation species choice. To assess these issues, we conducted a study in the Pearl River Delta region, Southern China, to quantify the responses of different functional group plants to urbanization. The Pearl River Delta region is urbanizing rapidly, and currently, it is also one of the largest urban areas worldwide (Cai et al., 2017). Four different functional groups (eight plant species totally) located in urban and suburban areas were selected and their adaptive strategies to urbanization were assessed via leaf traits, heavy metal content, photosynthesis, and N status. Results from this study will benefit our understandings of the physiological responses to the urbanizations and the species selections in urban green practice and managements.

## Materials and Methods

### Experimental Design and Sampling

Three different sites were selected to study the impacts of urbanization on plants (Supplementary Figure 1): the Urban Forest Park (UFP), the Suburban Forest Park (SFP), and the Rural Nature Reserve (RNR), which served as the three different treatments. The UFP (23°16′N, 113°22′E) was located in Baiyun District, Guangzhou, and served as the reference setting in this study, with a subtropical monsoon climate. In the UFP, the average annual temperature was 21.8°C and the average annual precipitation was 1,860 mm, with more than 80% of the rainfall being concentrated from April to September. The SFP (23°32′N, 113°45′E) and the RNR (24°07′N, 114°08′E) sites were approximately 70 and 150 km away from the UFP, respectively. They had similar subtropical monsoon climates: average annual temperatures were 20.7 and 19.5°C and average annual precipitations were 1,625 and 1,690 mm. The nitrogen (N) deposition and soil heavy metal concentration of the UFP were always the highest among the three sites, and more details are demonstrated in Supplementary Table 1. Eight common plant species at the three sites were selected: the herb species *Lophatherum gracile* and *Alpinia chinensis*, the shrub species *Ardisia quinquegona* and *Psychotria rubra*, the subcanopy species *Diospyros morrisiana* and *Cratoxylum cochinchinense*, and the canopy species *Engelhardtia roxburghiana* and *Schima superba*. Each treatment had eight replicated plants for each species at each site, with the total plant number being 192 (3 sites × 8 species × 8 replications of each species).

The healthy and mature leaves of the selected plant species were sampled for photosynthetic rate, light respiration rate, and other photosynthetic parameters measurement on sunny days (9:00–12:00 am) of August. After measurement, the leaves were collected to divide into three parts: one for leaf morphology and element concentration analysis; one for starches, sugars, and lipids determination; and the last one was frozen in liquid N_2_ and stored at −80°C for chlorophyll, ribulose-1, 5-bisphosphate carboxylase/oxygenase (Rubisco), and cell N contents determination.

### Leaf Traits Measurements

A total of 10 healthy leaves from each species were quantified with an electronic balance. Leaf thickness and surface area were measured with a thickness meter (Expolt, Expolt Ltd., PRC, Taiwan, China) and leaf area scanner (Biosciences, Lincoln, NE, United States), respectively. The leaves were dried at 105°C for 30 min and then at 70°C until they achieved a constant mass. The dry biomass was recorded.

The dried leaves were ground and passed through a 0.15-mm mesh sieve and digested with HClO_4_-H_2_SO_4_. The carbon (C) and N contents in the digested fractions were quantified with an elemental analyzer (Elementar Vario EL III, Hanau, Germany), and P content was assessed with the molybdenum antimony colorimetric method (Lu, 2000; Zhang et al., 2018). Abundances of ^13^C and ^15^N in the dried leaves were analyzed with an IsoPrime 100 mass spectrometer (Cheadle Hulme, United Kingdom), and intrinsic water use efficiency was also calculated based on the abundances of ^13^C (Osmond et al., 1980; Huang et al., 2016).

### Leaf Heavy Metal Concentration and Organic Matter Determination

Concentrations of heavy metals such as cadmium (Cd), chromium (Cr), copper (Cu), nickel (Ni), lead (Pb), and zinc (Zn) were determined by using an acid digestion method at atmospheric pressure. The dried leaf samples were placed in a 150-ml Erlenmeyer flask, mixed with hydrofluoric acid and nitric acid, and digested at 190°C. The mixtures were filtered and the concentrations of heavy metals in the solution were determined by the ICP-OES (Palo Alto, CA, United States). Data quality was also checked with national standard reference material (ESS-3) supplied by the China National Environmental Monitoring Centre. Heavy metal concentrations were expressed as an oven-dried mass.

The concentrations of starch in the leaves were determined according to the method of Smith and Zeeman (2006). Approximately, 0.2 g of dried leaf powder was extracted by 80% ethanol (80°C water bath) three times and the starch was quantified by measuring the hydrolyzed glucose content. Plant leaves were also extracted with a mixed solution (water, methanol, and chloroform), and then the contents of soluble sugar and soluble phenolic and lipid in the leaves were determined according to the method of Liu et al. (2016). After the extraction, the residue leaf samples were boiled in 3% hydrochloric acid (HCl) (v/v) for 3 h and the insoluble sugars in the supernatants were determined according to the method of Liu et al. (2016). The results of starch, soluble sugar, soluble phenol, lipid, and insoluble sugar contents were all converted and recorded as mg g^–1^ dry leaf.

### Leaf Photosynthetic Parameter Determination

Intact and healthy leaves were selected from the branches to construct photosynthetic light and carbon dioxide (CO_2_) response curves and determine stomatal conductance, intercellular CO_2_ concentration, and transpiration rate with a Li-6400XT portable photosynthetic system (Biosciences, Lincoln, NE, United States). In order to determine maximum photosynthetic rate, light component point, saturation irradiance, and respiration rate, the light response curves were constructed and fitted by using 14 different photosynthetic photon flux density (PPFD) gradients (1,500, 1,200, 1,000, 800, 500, 300, 200, 120, 100, 80, 60, 50, 20, and 0 μmol m^–2^ s^–1^); 400 μmol mol^–1^ CO_2_; 55–65% air humidity; 30°C leaf temperature; 1.5 kPa vapor pressure; and 30 min of induction time. The maximum carboxylation rate and maximum electron transport rate were quantified from the CO_2_ response curve with the methods of previous studies (Farquhar and von Caemmerer, 1982; Loustau et al., 1999). The CO_2_ curve was constructed under the conditions of 400, 300, 260, 200, 180, 150, 120, 100, 80, 60, 50, and 20 μmol⋅mol^–1^ CO_2_. Prior to this, the leaves were exposed to saturated PPFD for 30 min to reach full photosynthetic induction. Subsequently, stomatal conductance, intercellular CO_2_ concentrations, and transpiration rates were determined under saturated PPFD for each sample. Instantaneous water use efficiency was calculated by the ratio of the maximum photosynthetic rate and transpiration rate. Photosynthetic N or P use efficiency was calculated as follows (Hidaka and Kitayama, 2009):


$$\text{Photosynthetic}N\left(\mathrm{or}P\right)\mathrm{use}\mathrm{efficiency}=\frac{\mathrm{Maximum}\mathrm{photosynthetic}\mathrm{rate}}{totalN\left(orP\right)content/leafsurface}$$

### Leaf Chlorophyll Content and N Allocation Determination

Leaf chlorophyll content was determined within 24 h after fresh leaf sampling according to existing methods with minor modifications (Lichtenthaler and Buschmann, 2001; He et al., 2018). A leaf puncher was employed to cut three pieces (1 cm^2^) from the center blade of each fresh leaf and 30 pieces of the blade (10 leaves × 3 pieces of each leaf) were mixed together, ground, and extracted with 80% acetone in the dark. After filtering, chlorophyll a and b concentrations were calculated (Lichtenthaler and Buschmann, 2001; He et al., 2018).

Leaf Rubisco content was determined by the method of Makino et al. (1986). Approximately, 0.5 g of the leaf blade was ground, mixed with Tris-HCl buffer, and centrifuged at 4°C for 30 min. The supernatant was electrophoresed on sodium dodecyl sulfate (SDS)-polyacrylamide gel, stained with Coomassie Brilliant Blue for 12 h, and then decolorized. The gel band containing Rubisco was cut and eluted in a 50°C water bath. The Rubisco concentration was determined at 595 nm with a spectrophotometer, and bovine serum albumin was used as the standard. The N contents of cell walls were determined with the method of Harrison et al. (2009). Freeze-dried leaves (approximately 100 mg) were mixed with 15 ml buffer containing 1% polyvinyl pyrrolidine and centrifuged for 5 min. Precipitates were resuspended in 1% SDS buffer, heated to 90°C for 5 min, centrifuged for 5 min, washed with KOH, ddH_2_O, and ethanol, and dried at 80°C. The N content in the remaining matter was quantified with the elemental analyzer and recorded as the N contents of leaf cell walls. Area-based N content was calculated with the leaf N content to leaf surface ratio. The proportions of leaf N allocated to the photosynthetic system, Rubisco, carboxylation, bioenergetics, light-harvesting component, and cell wall were calculated according to the formula described in previous studies (Niinemets and Tenhunen, 1997; Xiao et al., 2018).

### Statistical Analysis

The two-way ANOVA was employed to determine the effect of urbanization and species on the test parameters and significant differences of urbanization for the same species among different sites were quantified by Duncan’s multiple range test. The Pearson’s correlation was used to quantify the linear correlations among the maximum photosynthetic rate and leaf area, leaf thickness, heavy metal concentration, and leaf N status. Leaf parameters were divided into four different groups: resource acquisition (specific leaf area, total N and P, maximum photosynthetic rate, and photosynthetic N use efficiency); adaption (water use efficiency, ^13^C, and ^15^N); defense (leaf thickness and soluble sugar and starch); and resistance (soluble phenol, liquid, and N allocation to cell wall) and they were analyzed with the confirmatory factor analysis to reveal their adaptive strategies to urbanization.

## Results

### Plant Leaf Morphological Traits

The effects of urbanization on leaf appearances were also dominated by the plant functional group differentiation. Although the herbs located at the different sites had approximately similar leaf biomasses (Supplementary Figure 2), dry leaf biomass of the shrub *A. quinquegona* located in the UFR was significantly (*p* < 0.05) lower than that in the SFR (Supplementary Figure 2b). There were also significant differences in the dry leaf biomasses of the subcanopies, with the lowest being in the species located in the UFP. The leaves of herbs or shrubs located in the UFR were thinner but larger compared to those of their suburban and rural counterparts (Supplementary Figures 2c,d), but the canopies had the opposite trends (thicker but smaller leaves). Meanwhile, the specific leaf area among the different sites had similar trends to those of leaf surface, with the values of herbs, shrubs, and subcanopies located in the UFP being significantly (*p* < 0.05) higher than those of their suburban and rural counterparts (Supplementary Figure 2e). However, relative to the UFP, the specific leaf areas of the canopies *E. roxburghiana* and *S. superba* in the RNR were 17.2 and 37.3% higher, respectively.

### Leaf Stoichiometry, Isotope Composition, and Intrinsic Water Use Efficiencies

**FIGURE 1:**
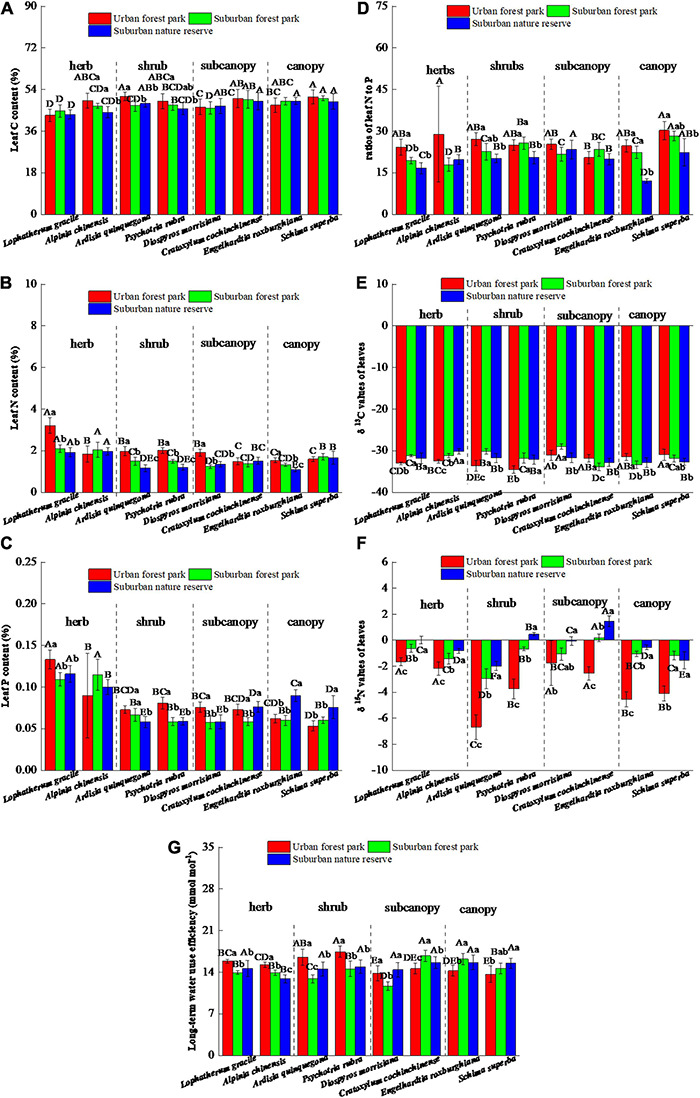
Effects of growth environment on **(A)** C content, **(B)** N content, **(C)** P content, **(D)** ratios of N to P, **(E)** δ ^13^C, **(F)** δ ^15^N, and **(G)** long-term water use efficiency of leaves of eight plant species. The lowercase letters reveal the significant differences (*P < 0.05*) of the same species among different growth environments, and the capital letters reveal the significant differences (*P < 0.05*) of different species in the same growth environment.

Compared with those grown in the RNR, the leaf C, N, and P contents of the shrub *A. quinquegona* in the UFP were 6.4, 68.7, and 25.4% higher, respectively, and the leaf C, N, and P contents of the shrub *P. rubra* grown in the UFP increased by 7.1, 67.0, and 37.7%, respectively (Figures 1A–C). However, the canopies of plants grown in the UFP had negligible variation in the leaf C contents but were significantly decreased in the leaf P contents relative to those grown in the RNR. The urban environment also tended to increase the ratios of leaf N to P (Figure 1D).

The eight plant species had significant differences in leaf δ^13^C values among the three sites. The leaf δ^13^C values of herbs and shrubs located in the UFP were significantly (*p* < 0.05) lower than their suburban counterparts, which are opposite to the situation in the canopies (Figure 1E). In the subcanopy species, the lowest leaf δ^13^C values were detected in plants located in the SFR and the RNR, respectively. However, the δ^15^N values of all the plants in the UFP were lowest among the three different sites and were significantly (*p* < 0.05) lower than those located in the RNR. The intrinsic water use efficiencies of the eight plant species were also significantly affected by urbanization and the herbs, shrubs, and canopies had different trends of the water use efficiencies among the three sites (urban > suburban in herbs and shrubs, but urban < suburban in canopies).

### Organic Matter Content and Heavy Metal Concentration in Leaves

The effects of urbanization on the organic matter in leaves were also dominated by the plant functional group differentiation (Supplementary Figure 3). Compared with the suburban and rural counterparts, the herbs and shrubs grown in the urban areas had significantly higher starch and soluble sugar contents. However, the differences between the plants grown in urban *vs.* suburban areas gradually diminished and even changed to be negative as the plant height increased (Supplementary Figures 3a,b). The leaf starch and soluble content levels of the canopy *E. roxburghiana* grown in the UFP were 80.0 and 58.4% of those grown in the SFP. For the soluble phenol and lipid contents, the numerical value of the herbs and shrubs of the urban were lower than those of the suburban, but the canopies results were reversed (Supplementary Figures 3c,d). There were inconsistent trends in the insoluble sugar content of plants at different sites and the canopy *E. roxburghiana* of the urban site contained the highest insoluble sugar content among all the samples (Supplementary Figure 3e).

**FIGURE 2:**
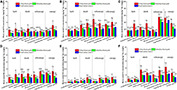
Effects of growth environment on heavy metal **(A)** Ni, **(B)** Cu, **(C)** Zn, **(D)** Pb, **(E)** Cd, and **(F)** Cr concentrations of leaves of eight plant species. The lowercase letters reveal the significant differences (*P < 0.05*) of the same species among different growth environments, and the capital letters reveal the significant differences (*P < 0.05*) of different species in the same growth environment.

Heavy metal concentrations in the leaves of all the eight plant species grown in urban areas were higher than those grown in the suburban and rural areas, with the sole exception of Cd content in *A. quinquegona* (Figure 2). Significant differences in leaf Ni concentrations were detected among *L. gracile*, *A. quinquegona*, and *E. roxburghiana* (Figure 2A), and the shrubs, subcanopies, and canopies all had significant differences in leaf Cu concentration at different sites (Figure 2B). Also, significant differences in Pb and Cr concentrations were found among their subcanopies at different sites (Figure 2C,D,F).

### Chlorophyll Content and Leaf Photosynthetic Properties

**FIGURE 3:**
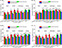
Effects of growth environment on contents of **(A)** chlorophyll a, **(B)** chlorophyll b, and **(C)** total chlorophyll and **(D)** ratio of chlorophyll a to b of eight plant species. The lowercase letters reveal the significant differences (*P < 0.05*) of the same species among different growth environments, and the capital letters reveal the significant differences (*P < 0.05*) of different species in the same growth environment.

Leaf chlorophyll, a content of the herbs and shrubs grown in the UFP, was significantly higher than those grown in the suburban and rural areas; however, the total chlorophyll differences between the urban and suburban plants changed from positive to negative with the plant height increases (Figures 3A,C). The *L. gracile*, *E. roxburghiana*, and *S. superba* species were significantly different in their leaf chlorophyll b contents among the three sites assessed (Figure 3B). Relative to the UFP, leaf chlorophyll b contents of the SFP grown plants were 13.7, 14.2, and 13.8% lower for the *L. gracile*, *E. roxburghiana*, and *S. superba*, respectively. The trends of chlorophyll a to b ratios among the three different sites were similar to those of leaf chlorophyll a (Figure 3D).

**FIGURE 4:**
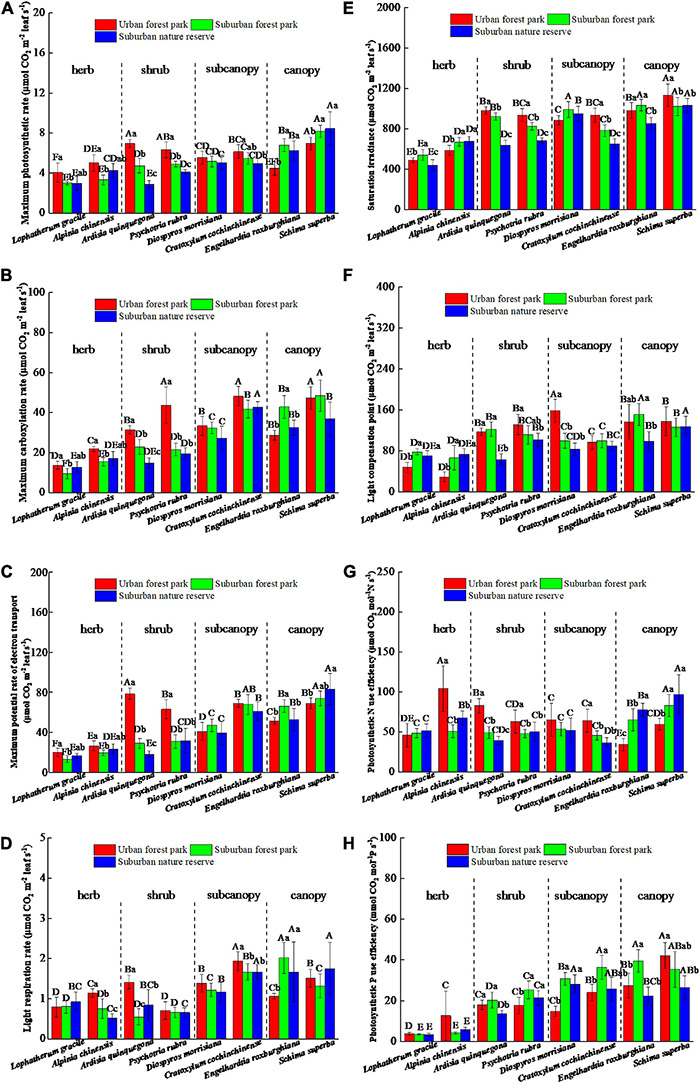
Effects of growth environment on **(A)** maximum photosynthetic rate, **(B)** maximum carboxylation rate, **(C)** maximum potential rate of electron transport, **(D)** light respiration rate, **(E)** saturation irradiance, **(F)** light compensation point, **(G)** photosynthetic N use efficiency, and **(H)** photosynthetic P use efficiencies of eight plant species. The lowercase letters reveal the significant differences (*P < 0.05*) of the same species among different growth environments, and the capital letters reveal the significant differences (*P < 0.05*) of different species in the same growth environment.

The effects of urbanization on the photosynthetic properties of leaves were dominated by the plant functional group differentiation (Figure 4). Relative to the suburban counterparts, the herbs and shrubs in the urban area had higher maximum photosynthetic rates, but the canopies of the suburban areas had significantly (*p* < 0.05) higher maximum photosynthetic rates than their urban counterparts (Figure 4A). The differences in maximum carboxylation rate and maximum potential rate of electron transport between the plants grown in the urban and suburban areas were initially negative and gradually increased with the plant functional groups (Figures 4B,C). The trend of light respiration rates of plants grown among the different sites was similar to those of the photosynthetic rate (Figure 4D). The saturation irradiance and light compensation point of the shrub located at the RNR were significantly lower than those of the UFP (Figures 4E,F). The photosynthetic N use efficiencies of the canopies significantly (*p* < 0.05) differed among the three sites and decreased in the order of RNR > SFP > UFP (Figure 4G). Plant photosynthetic P use efficiency increased with the plant height and the photosynthetic P use efficiencies of the subcanopies of plants grown in the UFP were significantly lower than those grown in the RNR (Figure 4H). The maximum photosynthetic rates of the different plant species were also positively correlated with leaf area (Table 1).

**TABLE 1:** Correlation coefficients and *P* levels of the Pearson’s correlation among maximum photosynthetic rate and plant external size, leaf appearance, heavy metal concentration, and N allocated into different components.

	***Lophatherum gracile***	***Alpinia chinensis***	***Ardisia quinquegona***	***Psychotria rubra***	***Diospyros morrisiana***	***Cratoxylum cochinchinense***	***Engelhardtia roxburghiana***	***Schima superba***
Height	0.356	0.016	0.018	0.076	0.366	0.481*	–0.376	–0.186
South-north crown width	0.125	0.441*	0.593**	0.816**	–0.270	0.252	0.374	–0.018
East-west crown width	0.111	0.177	0.436*	0.705**	–0.078	–0.100	0.386	0.204
Leaf thickness	–0.293	−0.612**	–0.266	–0.301	−0.474*	−0.443*	−0.651**	–0.197
Leaf surface	0.141	0.198	0.731**	0.514*	0.547**	0.254	0.369	0.409*
Specific area of leaves	0.534**	0.211	0.752**	0.682**	0.452*	0.630**	0.247	0.576**
Ni	0.536**	0.511*	0.619**	0.556**	0.055	0.594**	–0.211	−0.588**
Cu	0.473*	0.157	0.638**	0.630**	0.075	0.536**	–0.160	–0.149
Zn	–0.027	0.603**	0.547**	0.843**	0.384	0.334	–0.366	−0.437*
Pb	0.496*	0.066	0.445*	0.693**	0.539**	0.291	–0.250	–0.400
Cd	0.608**	0.146	–0.148	0.657**	0.340	0.279	−0.447*	–0.275
Cr	0.426*	0.158	0.566**	0.491*	0.358	0.270	−0.745**	−0.504*
Area-based leaf N	0.539**	–0.306	0.346	0.218	–0.371	−0.417*	–0.400	–0.206
Leaf N allocated to Rubisco	0.126	0.622**	0.831**	0.848**	0.618**	0.613**	0.731**	0.300
Leaf N allocated to bioenergetics	0.270	0.713**	0.850**	0.758**	0.663**	0.660**	0.657**	0.283
Leaf N allocated to light-harvesting components	−0.535**	0.483*	0.117	0.194	0.300	0.452*	0.370	0.160
Leaf N allocated to photosynthetic system	0.143	0.642**	0.846**	0.846**	0.639**	0.633**	0.733**	0.350
Leaf N allocated to carboxylation system	–0.068	0.604**	0.730**	0.774**	0.572**	0.610**	0.641**	0.330
Leaf N allocated to cell wall	−0.552**	–0.216	−0.500*	−0.619**	−0.418*	−0.468*	−0.411*	−0.628**

The bold values demonstrate the correlations are significant. *p < 0.05 and **p < 0.01.

Stomatal conductance, intercellular CO_2_ concentration, transpiration rate, and instantaneous water use efficiency were significantly but erratically affected by the urbanization (Supplementary Figure 4). The highest stomatal conductances of *L. gracile* and *A. chinensis* were both present in the UFP plants, which were significantly (*p* < 0.05) higher than those from plants grown in the SFP (Supplementary Figure 4a). The average intercellular CO_2_ concentration ranged from 206 to 270 μmol mol^–1^ and had erratic trends among all the plant species at the three sites (Supplementary Figure 4b). Significant differences in transpiration rates among the three sites were found among the herb, shrub, and subcanopy, but not found among the canopy samples (Supplementary Figure 4c). The responses of instantaneous water use efficiencies to the urbanization were dominated by the plant functional group differentiation. The instantaneous water use efficiency of the herbs, shrubs, and subcanopy plants located in the UFP were significantly (*p* < 0.05) lower than those grown in the RNR, which was opposite to the results of the canopies (Supplementary Figure 4d).

### Leaf N Allocated to Different Components

**FIGURE 5:**
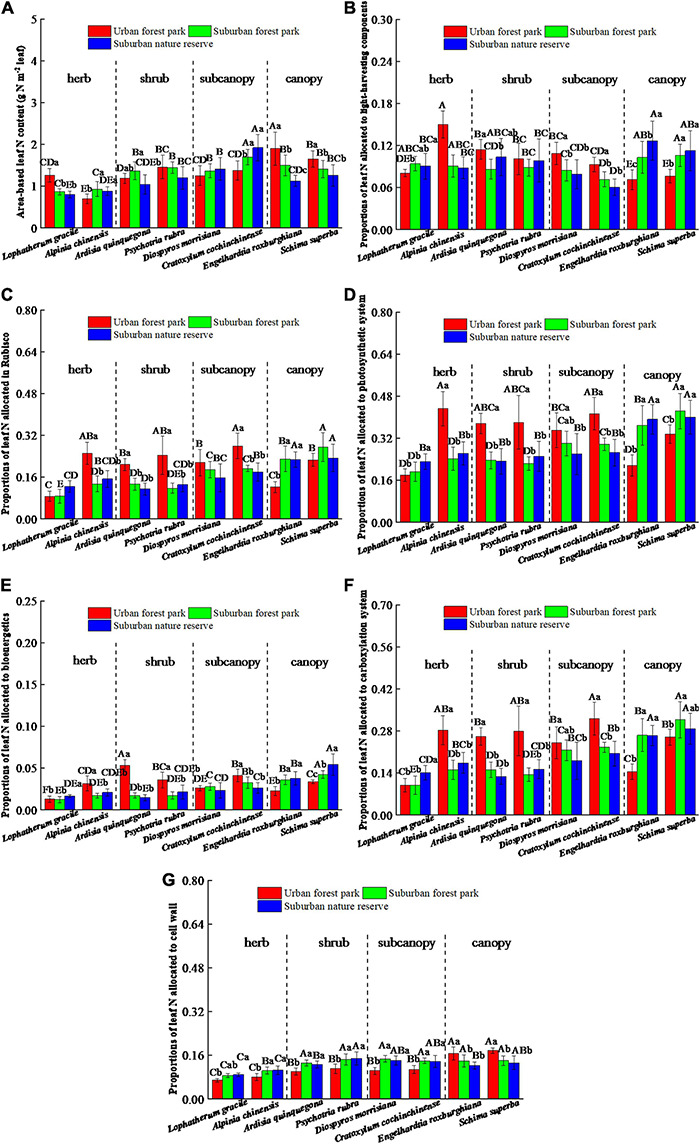
Effects of growth environment on **(A)** area-based leaf N content and proportions of leaf N allocated to the **(B)** Rubisco, **(C)** bioenergetics, **(D)** light-harvesting components, **(E)** photosynthetic system, **(F)** carboxylation system, and **(G)** cell wall of eight plant species. The lowercase letters reveal the significant differences (*P < 0.05*) of the same species among different growth environments, and the capital letters reveal the significant differences (*P < 0.05*) of different species in the same growth environment.

The leaf N allocated to the Rubisco, bioenergetics, and light-harvesting components had similar trends among the same plant species sampled at different sites (Figures 5B–D). Significant differences in leaf N allocated to the photosynthetic and carboxylation systems were all detected among the same species at different sites (Figures 5E,F). Among the three sites, the canopies of the urban area had the lowest proportions of leaf N in the photosynthetic and carboxylation systems. Responses of leaf N allocated to plant cell wall were significantly (*p* < 0.05) affected by the urbanization, with significant differences among four plant functional groups (Figure 5G). The proportions of leaf N allocated in the cell wall of the UFP herbs, shrubs, and subcanopies were significantly (*p* < 0.05) lower than those of the suburban and rural counterparts in stark contrast to the canopies.

### Adaptive Strategies and Plant Traits

**FIGURE 6:**
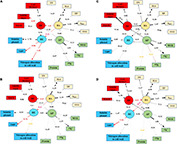
Confirmatory factor analysis on the trait loading values per integrated functions (RA, resource acquisition; AP, adaptation; DF, defense; RE, resistance) and relationships between the functions for canopy and understory plants in urban and suburban habitat: **(A)** canopy plants in urban area; **(B)** canopy plants in suburban areas; **(C)** understory plants in urban area; and **(D)** understory plants in suburban areas. The numbers in the line between the latent variable and its observed variables denoted loading values, and the numbers in the line between four latent variables showed regression coefficients.

The results showed that the canopies and subcanopies in the urban area suffered from environmental stress and they adapted to the stress via reducing resource acquisition, improving defense capacity, and enhancing resistance (Figure 6). For the canopies, the load of maximum photosynthetic rate in the urban area was approximately the same as those grown in the suburban area and a load of specific leaf area decreased from 1.29 when grown in the urban area to 0.78 when grown in the suburban area (Figures 6A,B). Urbanization may improve environmental adaptability and the defense of understory plants by improving resource acquisition ability (Figures 6C,D). Leaf ^13^C abundance (positive correlation) and water use efficiency (negative correlation) were the main characters related to plant adaptation. Soluble phenol and lipid concentrations may have contributed to resistance, with the load of soluble phenol < 1.0 under advantageous conditions.

## Discussion

### Stimulation of Urbanization on Plants

Our results showed that it was the plant functional groups that dominated the stimulation or inhibition function of urbanization on plant species and that urbanization appeared to have negative impacts on the canopies. Previous studies suggested that the CO_2_ concentration is higher in urban areas due to the proximity to additional emission sources. Since CO_2_ is the substrate of photosynthesis, it is unsurprising that elevated CO_2_ concentrations stimulate photosynthesis (Ainsworth and Rogers, 2007). Due to the heat island effect, the urban environment also has longer daytimes and higher air temperatures, promoting plant growth (Chapman et al., 2017). Moreover, relative to the urban areas, higher ozone (O_3_) concentration in suburban and rural areas has a negative impact on plant growth (Galant et al., 2012; Xie et al., 2016). Therefore, increased light resources, higher CO_2_ concentrations, atmospheric temperatures, and lower O_3_ concentrations in the urban area theoretically stimulated photosynthesis, leaf soluble sugar and starch accumulations, and whole plant growth. At the same time, Pretzsch et al. (2017) also confirmed that urbanization accelerated tree growth in the subtropical zone after the 1960s. The discrepancy may be due to the pressure of environmental pollutants originating from urbanization.

### Pressure of Urbanization on Plants

In this study, the negative correlations between heavy metal concentrations and maximum photosynthetic rates were all detected in the canopies (Table 1), which further implies that in the urban area, higher heavy metal concentrations in the leaves might offset the stimulating functions of urbanization on the canopies. It was also supported by previous studies, and Harrison (2018) also found that concentrations of traffic-generated pollutants in the urban background typically exceed the concentrations within suburban or rural areas. In the subtropical China area, all the concentrations of heavy metals in soils and dusts of the urban area were higher than their background values (Wei and Yang, 2010). Relative to the herbs and shrubs, the canopies were generally exposed to higher amounts of pollution due to the heights and larger external sizes, and thereby, the significant differences in leaf heavy metal concentration were more frequently detected in the canopies between the urban and the suburban areas (Figure 2). The parameters of chlorophyll fluorescence were more vulnerable to heavy metal stress and that heavy metal Cd inhibited at least two different targets in photosystem II (Rocchetta and Küpper, 2009; Rajkumar et al., 2013). The Rubisco activity and CO_2_ assimilation rate were also decreased in the presence of Cd (Cagno et al., 2008).

### Effects of Urbanization on Leaf N Proportions

The urbanization also significantly influenced the leaf cell N allocations (Figure 5). The N distribution in leaf cells reflected the physiological characteristics and survival strategies of plants under different environmental conditions (Warren and Adams, 2001; Ridenour et al., 2008). Leaf N is generally distributed into the cell wall, membrane, nucleus, chloroplast, mitochondrion, and some free compounds, and more than 50% of leaf N is allocated to chloroplasts for photosynthesis and energy transformation systems (Warren and Adams, 2001). The ratio of leaf N allocated into the cell wall indicated the tenacity of leaves, which is also a basic self-defense strategy of plants (Ridenour et al., 2008). Amidst harsh abiotic conditions, more leaf N would be allocated in the cell wall, resulting in a decline in photosynthetic system N (Ridenour et al., 2008). Consequently, the maximum photosynthetic rate was positively correlated with the proportions of leaf N allocated to the photosynthetic system but had negative correlations with the proportions of leaf N allocated in the cell wall (Table 1).

In this study, the average leaf N:P ratios on a mass basis ranged from 12.0 to 30.2 (Figure 1), which is in the range of average ratios reported in global datasets (Reich and Oleksyn, 2004; Hättenschwiler et al., 2008). Although urbanization tended to increase the leaf N contents and N:P ratios, Townsend et al. (2007) demonstrated that relative to the N content, plant productivity was generally limited by the P content. As observed for leaf N status, the δ^15^N value is a valuable parameter revealing long-term N sources of different plants (Hietz et al., 2011; Huang et al., 2016; Fu et al., 2020). Soil N saturation attributable to atmospheric N deposition influenced the plant δ^15^N value by enhancing nitrification and leaching (Hietz et al., 2011), and thereby, the decline in leaf δ^15^N of plants located in the urban was attributed to atmospheric N deposition. With reference to the studies conducted in different subtropical areas, atmospheric N deposition, including nitrogen oxides and ammonia, is frequently reported to be ^15^N depleted (Battipaglia et al., 2010; Chen et al., 2011; Fu et al., 2020). The N deposition could also lead to plant P limitation by soil acidification, which reduces available P via being fixed by activated iron and aluminum (Hou et al., 2018). For the canopies in this study, atmospheric N disposition of urbanization tended to increase N content but decreased P content (Figure 1), which might cause an imbalance of plant N and P and generate side effects on other nutrient absorptions. Moreover, the leaf N allocations also imply that relative to the suburban and rural environment, the urbanization positively stimulated the herb growth but inhibited the canopies.

### Adaptive Strategies of Plants

Apart from the inherent properties of the plant itself, plant growth is generally regulated by external environmental factors including light resource, temperature, fertilization, and pollutant stress. All these environmental factors had positive or negative effects on plant growth, and these effects could be accumulated, which discrepantly influence the physiological property and growth of plants. The urban, suburban, and rural areas had a series of differences in these environmental factors. Relative to the suburban and rural counterparts, the urban environment differs in many aspects that may confound the responses of nutrient supplies and pollution. Consequently, plants appear to adopt different strategies, including photosynthetic rates and leaf N and P allocation, to adapt to these environments (Figure 6).

Photosynthesis is the primary assimilation process for most plants, which is also one of the key parameters affecting plant adaptive strategies to different environments (Houborg et al., 2013; Schliep et al., 2013). The plant photosynthetic capacity is quantified with the maximum photosynthetic rate under suitable conditions. In plants, the chlorophyll is responsible for absorbing and converting light energy, and thereby, the changes in chlorophyll contents directly demonstrated the advantages and disadvantages of urbanization on plant physiology at a molecular level (Wasielewski et al., 1981; Papageorgiou and Govindjee, 2004; Schliep et al., 2013). The primary pigment for plant photosynthesis is chlorophyll a and the chlorophyll b generally serves as an accessory pigment (Wasielewski et al., 1981; Schliep et al., 2013). The chlorophyll a absorbs light from the orange-red and violet-blue electromagnetic spectrum and the leaf contained more chlorophyll content with a higher efficient capture of photons and photosynthetic rate (Papageorgiou and Govindjee, 2004; Schliep et al., 2013). In this study, relative to the suburban and rural area, urbanization had positive effects on the total chlorophyll contents of the herbs and shrubs, namely, herbs and shrubs changed in their physiologies to adapt to stimulating roles of urbanization (Figure 6). For photosynthesis, light as an energy resource is essential to sustain plant growth and ensure long-term survival (Craine and Dybzinski, 2013). Compared to the suburban and rural environment, more artificial light sources exist in urban areas and it has been proved that artificial light could delay leaf senescence (Zhang et al., 2015).

## Conclusion

Compared with the suburban and rural counterparts, the herbs and shrubs in the urban area had significantly higher starch and soluble sugar contents, but the canopies had significantly lower soluble sugar contents. Urbanization increased leaf heavy metal concentration and N content but decreased leaf δ^15^N. The urbanization enhanced the maximum photosynthetic rate and total chlorophyll contents of the herbs and shrubs but decreased the maximum photosynthetic rate and total chlorophyll contents of the canopies. The proportions of leaf N allocated in the cell wall of urban herbs, shrubs, and subcanopies were significantly lower than those of the suburban and rural counterparts in stark contrast to the canopies. This study also demonstrated that plant functional groups dominated responses of adaptive strategies to urbanization and that urbanization tended to stimulate the herbs but inhibit the canopies. The canopies and subcanopies in the urban area suffered from environmental stress, and they adapted to the stress via reducing resource acquisition, improving defense capacity, and enhancing resistance. However, urbanization could improve environmental adaptability and the defense of understory plants by improving resource acquisition ability.

## Data Availability Statement

The original contributions presented in the study are included in the article/Supplementary Material, further inquiries can be directed to the corresponding author/s.

## Author Contributions

YX, SL, MZ, ZX, FT, XS, TL, TZ, and ZW were all involved in conceptualizing, designing, and implementing the project. YX and MZ prepared the manuscript. ZX and RF helped to revise the manuscript. YX carried out all the data collection and drafted the manuscript. All authors contributed to the article and approved the submitted version.

## Conflict of Interest

The authors declare that the research was conducted in the absence of any commercial or financial relationships that could be construed as a potential conflict of interest.

## Publisher’s Note

All claims expressed in this article are solely those of the authors and do not necessarily represent those of their affiliated organizations, or those of the publisher, the editors and the reviewers. Any product that may be evaluated in this article, or claim that may be made by its manufacturer, is not guaranteed or endorsed by the publisher.
