# Supplementary material for: Plant Functional Groups Dominate Responses of Plant Adaptive Strategies to Urbanization
Source: Front Plant Sci. 2021 Nov 30;12:773676. doi: 10.3389/fpls.2021.773676 (PMC8669269; doi:10.3389/fpls.2021.773676)
Supplement: Supplementary file 1 [file Table_1.DOC]

**Plant functional groups dominate responses of plant adaptive strategies to urbanization**

*Yihua Xiao ^1^, Shirong Liu ^2^, Manyun Zhang ^3, 4^,* *[[1]](#footnote-2)*, Fuchun Tong ^5^, Zhihong Xu ^4^, Rebecca Ford ^4^，Tianlin Zhang ^1^, Xin Shi ^1^, Zhongmin Wu ^1^, Tushou Luo ^1^*

1. Research Institute of Tropical Forestry, Chinese Academy of Forestry, Guangzhou 510520, China

2. The Research Institute of Forest Ecology, Environment and Protection, Chinese Academy of Forestry, Beijing 100091, China

3. College of Resources and Environment, Hunan Agricultural University, Changsha 410128, China

4. Environmental Futures Research Institute, School of Environment and Science, Griffith University, Brisbane 4111, Australia

5. College of Forestry and Landscape Architecture，South China Agricultural University，Guangzhou 510642, China

**Fig. S1** The three experimental sites

**Table S1** The basic background information of the three experiment site.

**Fig. S2** Effects of growth environment on (a) fresh biomass, (b) dry biomass, (c) thickness, (d) surface and (e) specific area of leaves of eight plant species. The lowercase letters reveal the significant differences (*P < 0.05*) of the same species among different growth environments, and the capital letters reveals the significant differences (*P < 0.05*) of different species at the same growth environment.

**Fig. S3** Effects of growth environment on (a) starch, (b) soluble sugar, (c) soluble phenol, (d) lipid and (e) insoluble sugar contents of leaves of eight plant species. The lowercase letters reveal the significant differences (*P < 0.05*) of the same species among different growth environments, and the capital letters reveals the significant differences (*P < 0.05*) of different species at the same growth environment.

**Fig. S4** Effects of growth environment on (a) stomatal conductance, (b) intercellular CO_2_ concentration, (c) transpiration rate and (d) instantaneous water use efficiency of leaves of eight plant species. The lowercase letters reveal the significant differences (*P < 0.05*) of the same species among different growth environments, and the capital letters reveals the significant differences (*P < 0.05*) of different species at the same growth environment.


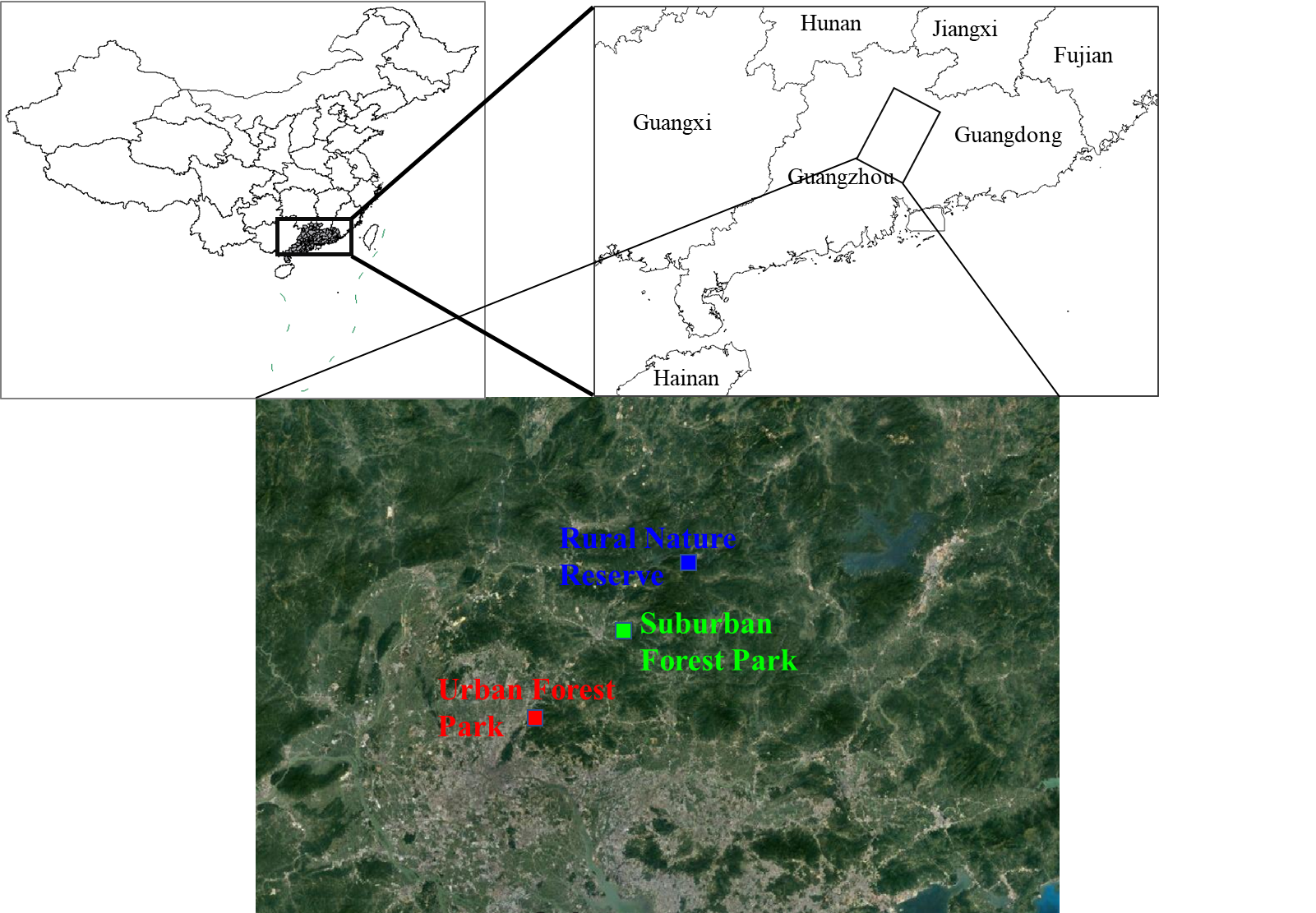


**Fig. S1** The three experimental sites

| Sites | Mean atmospheric temperature | Mean precipitation | N deposition | Zn concentration in 0-10 cm soil | Mn concentration in 0-10 cm soil | Pb concentration in 0-10 cm soil | Cu concentration in 0-10 cm soil | Cr  concentration in 0-10 cm soil | Ni  concentration in 0-10 cm soil |
| --- | --- | --- | --- | --- | --- | --- | --- | --- | --- |
|  | (℃) | (mm) | (kg N ha^-1^ yr^-1^) | (mg kg^-1^) | (mg kg^-1^) | (mg kg^-1^) | (mg kg^-1^) | (mg kg^-1^) | (mg kg^-1^) |
| Urban forest park | 21.8 | 1860 | 21.9 | 196.5 | 103.2 | 74.9 | 21.8 | 29.6 | 5.6 |
| Suburban forest park | 20.7 | 1625 | 18.1 | 105.3 | 67.4 | 49.4 | 13.5 | 16.3 | 3.8 |
| Rural nature reserve | 19.5 | 1690 | 16.2 | 97.6 | 77.0 | 45.7 | 10.7 | 19.2 | 4.5 |

**Table S1** The basic background information of the three experiment site.


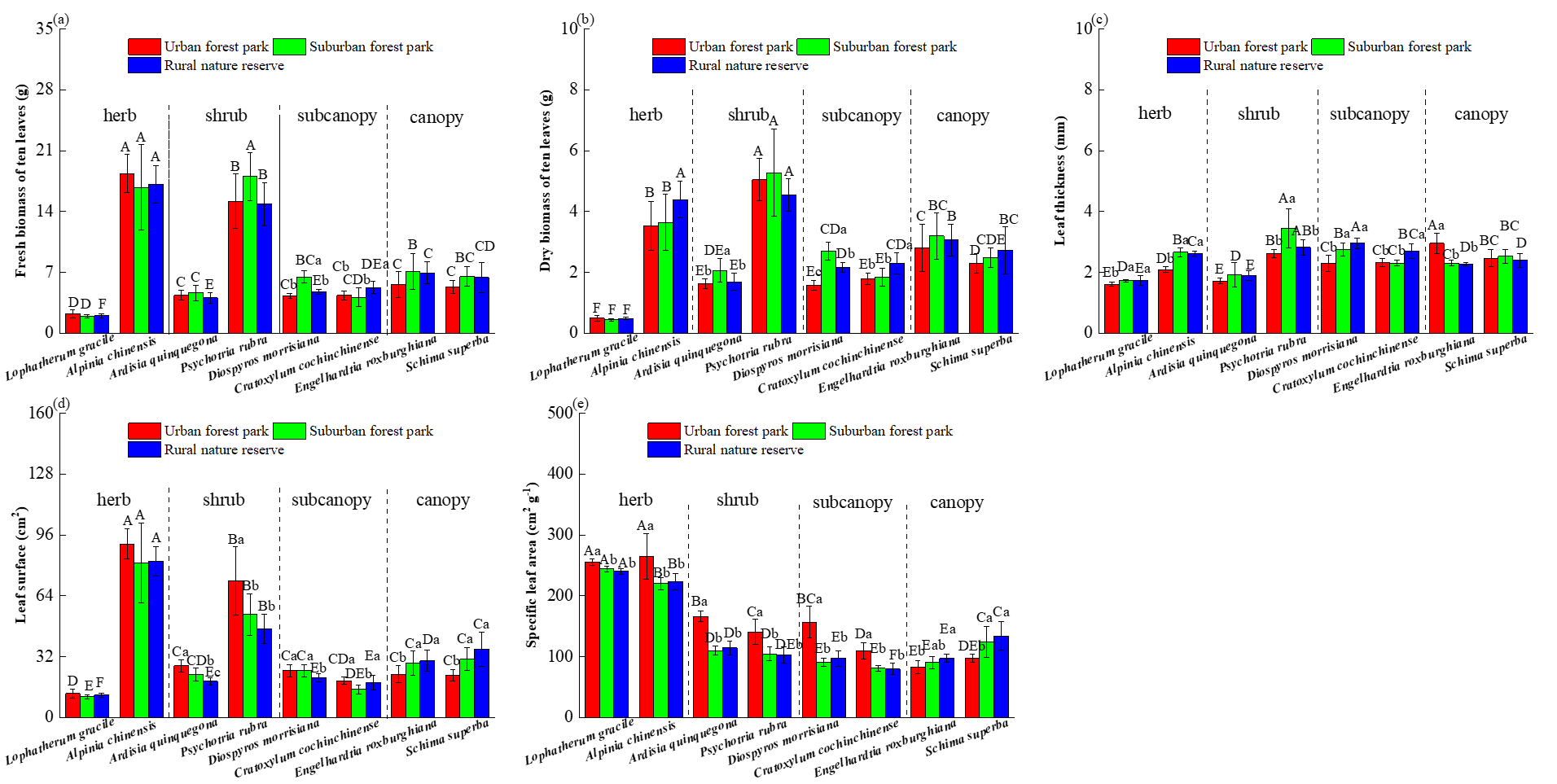


**Fig. S2** Effects of growth environment on (a) fresh biomass, (b) dry biomass, (c) thickness, (d) surface and (e) specific area of leaves of eight plant species. The lowercase letters reveal the significant differences (*P < 0.05*) of the same species among different growth environments, and the capital letters reveals the significant differences (*P < 0.05*) of different species at the same growth environment.


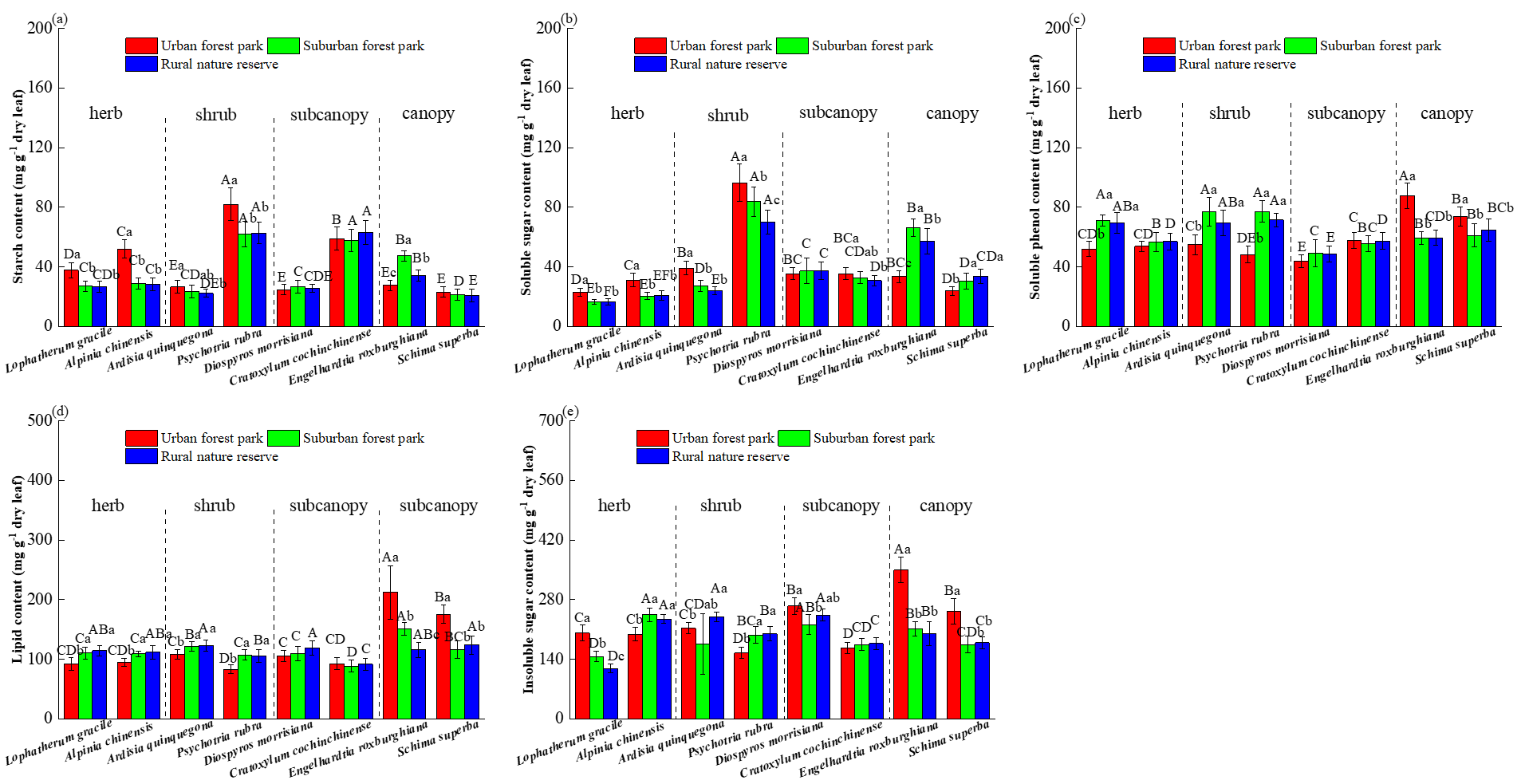


**Fig. S3** Effects of growth environment on (a) starch, (b) soluble sugar, (c) soluble phenol, (d) lipid and (e) insoluble sugar contents of leaves of eight plant species. The lowercase letters reveal the significant differences (*P < 0.05*) of the same species among different growth environments, and the capital letters reveals the significant differences (*P < 0.05*) of different species at the same growth environment.


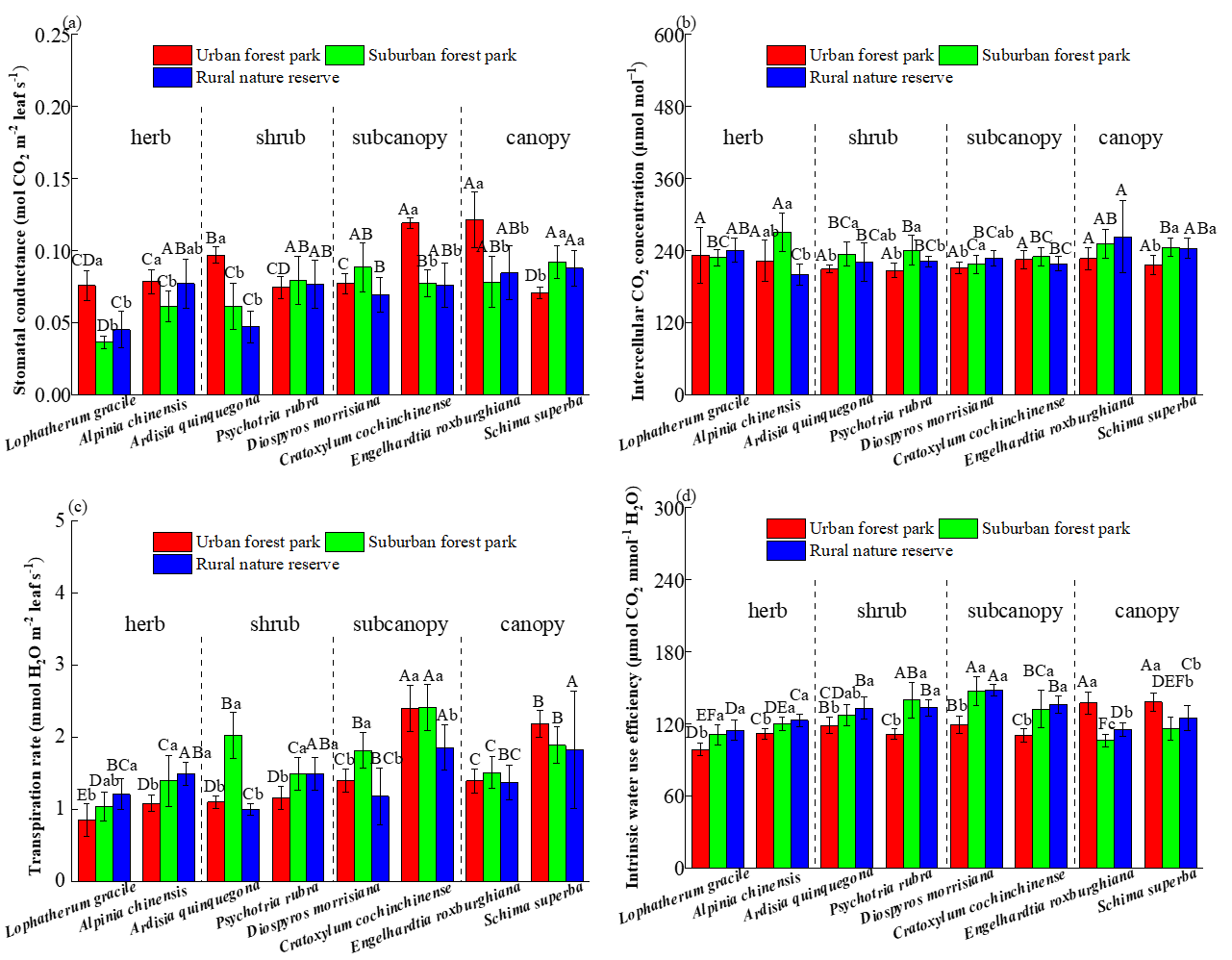


**Fig. S4** Effects of growth environment on (a) stomatal conductance, (b) intercellular CO_2_ concentration, (c) transpiration rate and (d) instantaneous water use efficiency of leaves of eight plant species. The lowercase letters reveal the significant differences (*P < 0.05*) of the same species among different growth environments, and the capital letters reveals the significant differences (*P < 0.05*) of different species at the same growth environment.

1. ^^ Corresponding author, E-mail: manyunzhang@126.com [↑](#footnote-ref-2)
